# Photocatalytic and Antibacterial Activity of CoFe_2_O_4_ Nanoparticles from *Hibiscus rosa-sinensis* Plant Extract

**DOI:** 10.3390/nano12203668

**Published:** 2022-10-19

**Authors:** Lakshmi Velayutham, C. Parvathiraja, Dhivya Christo Anitha, K. Mahalakshmi, Mary Jenila, Fatmah Ali Alasmary, Amani Salem Almalki, Amjad Iqbal, Wen-Cheng Lai

**Affiliations:** 1 Department of Physics, St. Xavier’s College (Autonomous), Manonmaniam Sundaranar University, Palayamkottai, Tirunelveli 627002, Tamilnadu, India; 2 Department of Physics, Manonmaniam Sundaranar University, Tirunelveli 627012, Tamilnadu, India; 3 Chemistry Department, College of Science, King Saud University, Riyadh 11451, Saudi Arabia; 4 Department of Advanced Materials & Technologies, Faculty of Materials Engineering, Silesian University of Technology, 44-100 Gliwice, Poland; 5 Bachelor Program in Industrial Projects, National Yunlin University of Science and Technology, Douliu 640301, Taiwan; 6 Department of Electronic Engineering, National Yunlin University of Science and Technology, Douliu 640301, Taiwan

**Keywords:** biosynthesis, CoFe_2_O_4_, nanoparticles, photocatalysis, degradation

## Abstract

Biogenic CoFe_2_O_4_ nanoparticles were prepared by co-precipitation and *Hibiscus rosa sinensis* plant leaf was used as a bio-reductant of the nanoparticle productions. The biosynthesized CoFe_2_O_4_ nanoparticles were characterized by XRD, FTIR, UV, VSM, and SEM via EDX analysis. The cubic phase of biosynthesized CoFe_2_O_4_ nanoparticles and their crystallite size was determined by XRD. The Co-Fe-O bonding and cation displacement was confirmed by FTIR spectroscopy. The presence of spherically-shaped biosynthesized CoFe_2_O_4_ nanoparticles and their material were confirmed by SEM and TEM via EDX. The super-paramagnetic behaviour of the biosynthesized CoFe_2_O_4_ nanoparticles and magnetic pulse was established by VSM analysis. Organic and bacterial pollutants were eradicated using the biosynthesized CoFe_2_O_4_ nanoparticles. The spinel ferrite biosynthesized CoFe_2_O_4_ nanoparticles generate radical and superoxide ions, which degrade toxic organic and bacterial pollutants in the environment.

## 1. Introduction

Ferrite nanoparticles have found a wide range of applications due to their electrical and magnetic properties and their uses in the biomedical field [1]. They have significant potential for use in a variety of applications, including wastewater treatment, catalysis, biomedicine, and magnetic energy storage, thanks to their special composition and microstructure [2]. Environmental toxicity results from the chemical synthesis of ferrite nanoparticles and the non-uniform particle size and porosity that results from their physical synthesis. [3] To overcome these difficulties in synthesis, biological technologies provide a more environmentally friendly option over chemical and physical approaches. [4] Green chemistry is inspired by nature, specifically plants, yeast, fungi, and bacteria. The incorporation of green chemistry concepts is a critical topic in nanoscience research. One of the most intriguing spinel ferrites is inverse spinel cobalt ferrite (CoFe_2_O_4_), which has high physical and chemical stability, high anisotropy and saturation magnetization, and tunable coercivity [5,6], making it a good candidate for biological applications [6,7]. Traditional Chinese medicine has long used *hibiscus rosa-sinensis* as a medicinal herb to treat a wide range of illnesses. Approximately 50 years have passed since its antibacterial effect was first discovered [8]. In its chemical components, this plant contains phenolic and organic acids such as homogentisic, hibiscus, malic, succinic, citric, succinic, and succinic acids. Other flavonoids exist, including their glycosides, gossypetin, luteolin, and quercetin. The vibrant colour of the flowers is a result of anthocyanins. The mineral content varies according to the species, place of origin, age, and colour. The antioxidant and antibacterial action is brought on by flavonoids and total phenolic substances [9]. *Hibiscus rosa sinensis* has numerous antioxidant products such as beta-carotene, vitamin C, and anthocyanin. These products are involved in reactions involving antifertility, skin protection, hair strengthening, cardiovascular enrichment, restricting premature aging, and increasing weight loss. Biomolecule-covered nanoparticles can promote particle charge and increase the separation between charged products. Green synthesis of ferrite nanoparticles is a fine and easy synthesis methods compared to hydrocarbonate or oxalate co-precipitation methods. Green synthesis is an easy synthesis method, and the derivatives do not moderate the ecosystem. Cobalt ferrite nanoparticles have been shown to have antibacterial efficacy against multidrug-resistant bacterial strains in recent investigations [10]. It is well known that the synthesis process has a significant impact on the magnetic ferrite nanoparticles’ composition, structure, and shape, and implicitly on their characteristics. For this reason, researchers are concentrating on creating nanoparticles with predictable sizes and morphologies [11]. The possibility of reducing the chances of harmful compounds is provided by the biosynthetic approach to the synthesis of nanoparticles using plant extracts from leaves, flowers, roots, or seeds. A range of metabolites, including carbohydrates, polysaccharides, phenols, amino acids, and vitamins, are present in the plant extracts and may be released as a result [11,12,13,14]. These metabolites may serve as capping agents, reducing agents, or stabilizing agents for “catching” the metal ions. The present research summarizes the current status of ferrite nanoparticles using green synthesis techniques, their properties, and their applications. Furthermore, several plant extracts are used in the green synthesis of metal oxide nanoparticles, including Aloe vera leaves, ginger root, and *Hibiscus rosa-sinensis* leaf. While numerous methods are available to synthesize cobalt ferrite nanoparticles, green production of cobalt ferrite nanoparticles is an ecologically friendly and easy method of production. Other methods require chemical substrates, long reaction times, and well equipped labs and instrumentation, and their output production is very low. Moreover, other methods produce primary and releasing secondary toxic gases, and their outlets are very noxious for the environment. Green methods of nano-production do not require high end lab facilities, long running times, or toxic chemicals, which is mostly appreciable for large-scale industrial productions [15,16,17,18,19,20,21]. Discharge of dyestuffs is one of the most deadly pollutants produced by many different industries. Every day, new dyes are formulated and commercialized for various applications. Aquatic system and soil systems are directly or indirectly affected by release of unmodified dyestuffs. The released dyestuffs penetrate to the soil system, which does not allow light penetration and demotivates photosynthesis, which restricts the life cycle. Methylene Blue (MB) is one such toxic and carcinogenic dye, and its easily soluble and highly stable cationic dyes. Direct and indirect exposure of MB dye approaches are very harmful and a major threat to safety, the environment, and microorganism evolution [22,23,24,25,26,27,28,29]. Additionally, ferrites have important photo-catalytic properties that are useful for many industrial processes, including the oxidative dehydrogenation of hydrocarbons [17], oxidation of compounds [18], and decomposition of alcohols and peroxides [15]. Ferrites’ properties are significantly influenced by the location, kind, and amount of metal used in their construction [19]. For instance, the redox properties of ferrites are considerably changed when transition metals such as Co^+2^ [20] are substituted into the spinel lattice. Ferrites feature a band gap that can absorb visible light in addition to having a spinel crystal structure, which boosts efficiency by adding more catalytic sites via the crystal lattice [21]. A thorough review of the literature shows that there are currently relatively few studies that have examined pure metal ferrites as catalysts for the oxidative degradation of organic contaminants and dyes [22]. The first investigation of the manufacture of spinel copper ferrite (CuFe_2_O_4_) using hibiscus flower extract was reported in 2015 by Manikandan et al. [23]. Based on the above-mentioned discussions of the advantages of using ferrites as part of our ongoing research project [24] on nanocatalysts and their uses, the current aims of this work are to synthesize cobalt ferrite nanoparticles (CoFe_2_O_4_) through environmentally friendly methods using a methanolic extract of *Hibiscus rosa-sinensis* leaf and to test the antimicrobial activity of CoFe_2_O_4_ nanoparticles.

## 2. Materials and Methods

### 2.1. Materials

The synthesized chemicals were purchased from HiMedia-Mumbai, India. Cobalt (II) nitrate hexahydrate and Ferric nitrate nonahydrate were used to synthesize the CoFe_2_O_4_ NPs. All chemicals used were of analytical reagent (AR) grade, and there was no extra modification during the synthesis time. *Hibiscus rosa-sinensis* used as an alternative reducing material, as the plant nutrients are able to initiate the action of capping, stabilization, and reduction of source materials. Double-distilled water was used to process and further synthesize the nanoparticles.

### 2.2. Preparation of Hibiscus rosa-sinensis Leaf Extract

The 100 mg of *Hibiscus rosa-sinensis* fresh leaves were collected from St. Xavier college campus at Tirunelveli, Tamilnadu, India. The collected leaves were washed with tap water and processed with double-distilled water; 100 mg of leaf extract was mixed with 100 mL double-distilled water, then stirred for a time along with heating conditions. The mixed solution was filtered using Whatman no. 1 filter paper and kept in −4 °C conditions for further evaluation.

### 2.3. Synthesis of CoFe_2_O_4_ NPs

**Scheme 1:**
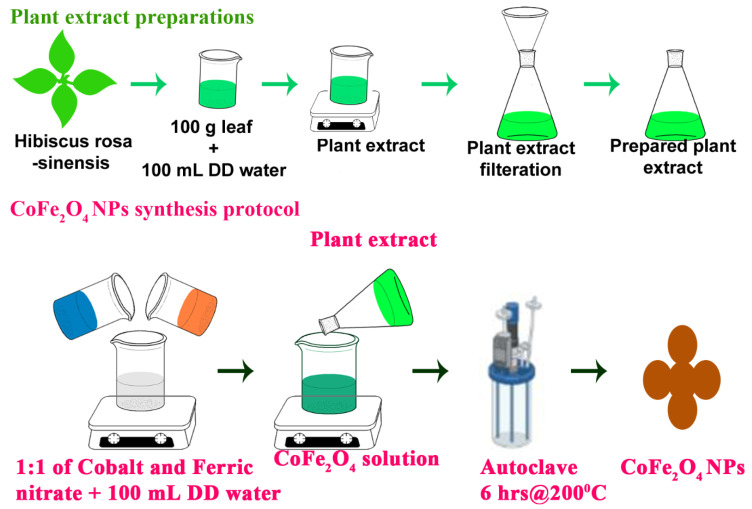
Synthesis protocol of CoFe_2_O_4_ NPs.

An equal mole ratio (1:1) of cobalt nitrate nonahydrate and ferric nitrate hexahydrate was used in the synthesis process of CoFe_2_O_4_ NPs. Cobalt and ferric materials were dissolved in 100 mL double-distilled water and stirred (temperature = 60 °C) for 3 h to attain a homogeneous solution. The combined homogeneous solution produced a light brown colour. The stored 10 mL leaf extract was poured into the cobalt and ferrite mixed solution and stirred for 1 h (temperature = 60 °C). The colour turned to a dark brown, indicating the reduction of nitrate and the size of the particles. The obtained dark brown solution was transferred into an autoclave and heated to 200 °C for 6 hrs. Finally, the obtained samples were filtered using Whatman no. 1 filter paper and calcined at 300 °C for 2 h. The synthesized CoFe_2_O_4_ NPs were stored for further characterization. The synthesized CoFe_2_O_4_ NPs protocol is presented in Scheme 1.

### 2.4. Characterization of CoFe_2_O_4_ NPs

The synthesized CoFe_2_O_4_ NPs structural integrity was determined using an X-ray diffractometer (X-pert Pro, PANalytical B.V., Overijssel, The Netherlands). The functional group and chemical bonds of the title materials were observed from FTIR spectroscopy (Perkin Elmer, Waltham, MA, USA). The optical stability and configurations were constructed from UV-DRS (Shimadzu-2700, Kyoto, Japan). The surface modification and information were monitored via FESEM analysis and TEM (TITAN, Julich, Germany), and their existing materials were identified from EDX spectroscopy (Carl Zeiss, Jena, Germany).

### 2.5. Antibacterial Activity

The antibacterial stability of synthesized CoFe_2_O_4_ NPs was evaluated by the disc diffusion method. The two different bacterial strains (*S. aureus*-9779 and *E. coli*-745) were used in this experiment. The bacterial culture was prepared by Muller–Hinton Agar (MHA). The active bacterial culture was spread over sterilized Petri plates. The paper discs were immersed in synthesized CoFe_2_O_4_ NPs (10 mg) and loaded with active bacteria culture (1 × 10^8^ CFU/mL). The obtained Petri plates were stored for incubation for 24 h at 36 °C. The bacterial inactivation capacity of synthesized CoFe_2_O_4_ NPs was demonstrated by the zone of inhibition over the Petri plates, with the measurement was calculated on a mm scale.

### 2.6. Photocatalytic Dye Degradation Activity

The visible light photocatalytic activity of CoFe_2_O_4_ NPs and commercial photocatalyst P-25 was examined using methylene blue (MB) dye under visible light irradiation (Xenon lamp; wavelength = 400 nm). Concentrations of 10 ppm MB dye were mixed with 10 mg CoFe_2_O_4_ NPs and stirred for 30 min, then kept in dark conditions to reach adsorption–desorption equilibrium. Then, the combined solution was placed in light conditions and kept out every 30 min to measure the absorbance spectrum. The withdrawn samples were centrifuged at 10,000 rpm for 5 min to eliminate the catalyst from the dye solution. The dye degradation percentage was calculated by the following equation.

$$\mathrm{Dye}\;\mathrm{degradation}\;\mathrm{percentage}\;(\%)=\frac{C-Co}{Co}\;\times \;100$$

*C*—reactive dye absorbance value every 30 min.

*Co*—Initial dye absorbance value at 0 min.

## 3. Result and Discussions

### 3.1. XRD Analysis

**Figure 1:**
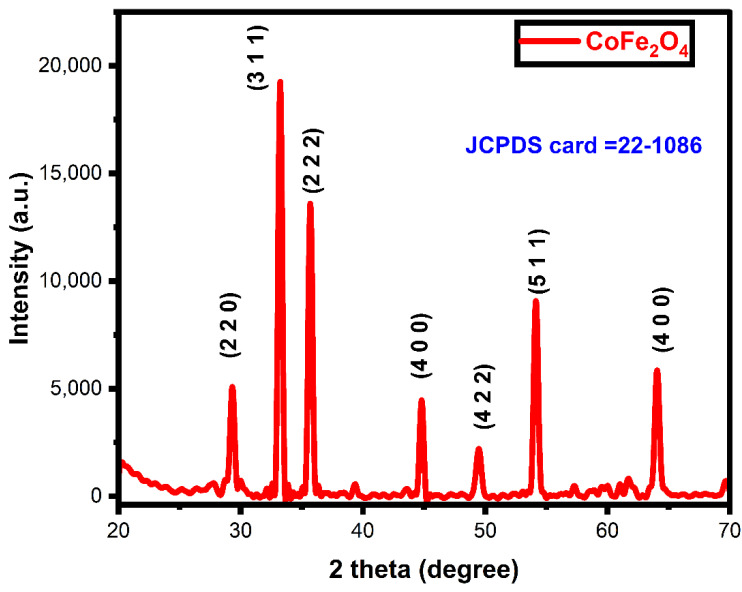
XRD pattern of biosynthesized CoFe_2_O_4_ nanoparticles.

The X-ray diffraction of biosynthesized CoFe_2_O_4_ nanoparticles is presented in Figure 1. The diffraction pattern shows the crystallinity and phase structure of biosynthesized CoFe_2_O_4_ nanoparticles. The obtained biosynthesized CoFe_2_O_4_ nanoparticles 2-theta values coincide with standard cubic phase CoFe_2_O_4_ JCPDS card number 22-1086 [25,26], and their (hkl) plane values are (220), (311), (222), (400), (422), (511), and (400) respectively. The cubic spinel ferrites of biosynthesized CoFe_2_O_4_ nanoparticles crystallite size were calculated by the Debye-Scherrer formula [25], with their calculated size being 24 nm. The spinel ferrites were formed over the cobalt materials due to their atomic radius (200 pm). The iron-coupled cobalt materials are located in tetrahedral sites and the rest of the iron materials are positioned in octahedral sites [26,27]. The combination of cobalt and iron metals increased the oxygen vacancy, which improved the nanostructure formations. The nanoparticles’ growth was well established and refined by lattice oxygen and their bonding between the cobalt and iron materials. The small crystallite size and cubic spinel ferrites of biosynthesized CoFe_2_O_4_ nanoparticles have increased degradation activity against dyes and pathogens.

### 3.2. FTIR Analysis

**Figure 2:**
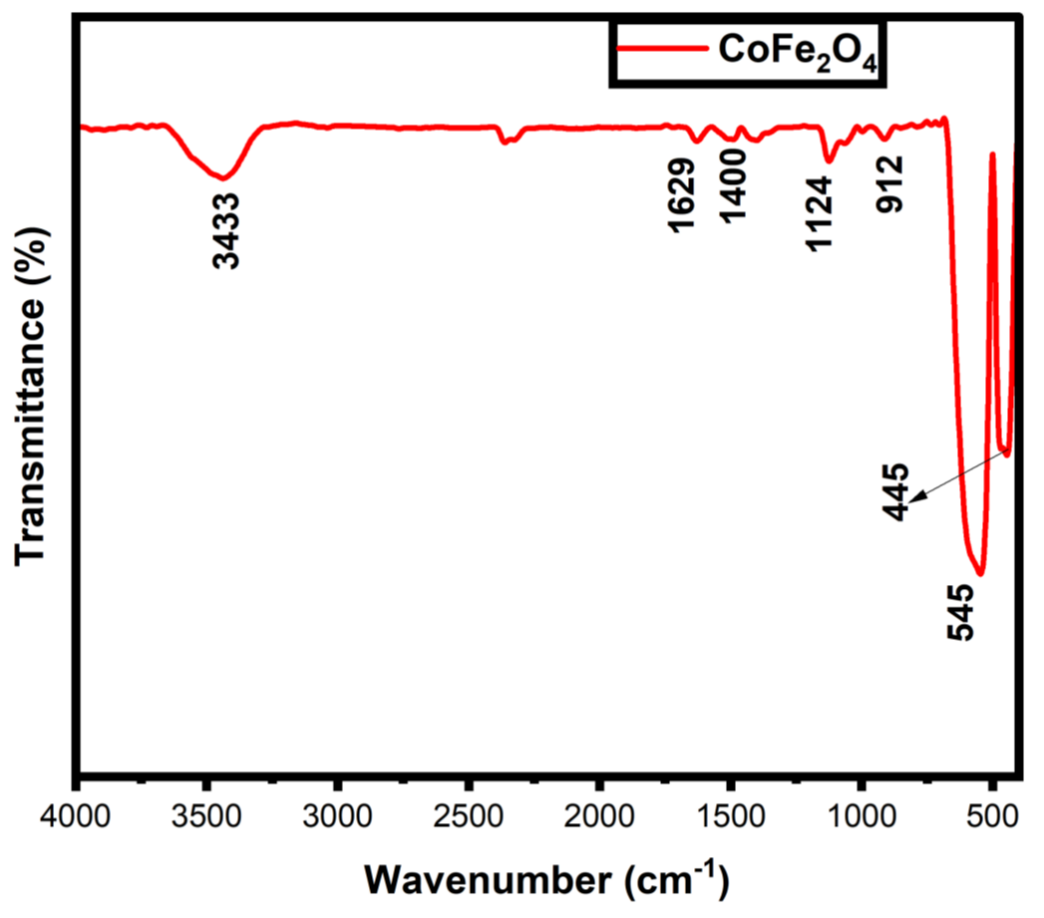
FTIR spectrum of biosynthesized CoFe_2_O_4_ nanoparticles.

The biosynthesized CoFe_2_O_4_ nanoparticles functional groups and their reductions and stabilization of the title compounds were elucidated by FTIR spectroscopy; their FTIR spectrum is shown in Figure 2. The broad peak at 3433 cm^−1^ indicates -OH stretching on the surface of the molecules [28]. The peaks at 1629 cm^−1^,1400 cm^−1^, and 1124 cm^−1^ represent the amide, amine, and carbon groups that are involved in the reduction, capping, and stabilization of the CoFe_2_O_4_ nanoparticles [29,30]. The Fe-O-Co interface and their bridge formations were attained from the phenolic compounds of the plant extract. The tetrahedral sites of Fe and Co [31,32] were confirmed by the peaks of 912 cm^−1^, 545 cm^−1^, and 445 cm^−1^. The plant compounds induced spinel ferrite formation and motivated enhanced degradation and bio-activities.

### 3.3. UV-Visible Analysis

**Figure 3:**
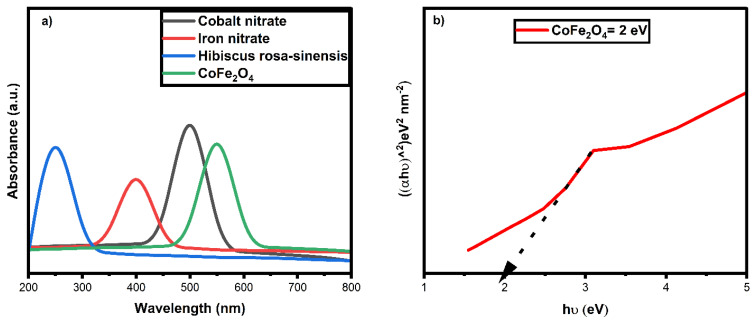
UV-Visible cobalt nitrate, iron nitrate, plant extract, and absorbance (**a**) and bandgap spectrum (**b**) of the biosynthesized CoFe_2_O_4_ nanoparticles.

The pure cobalt nitrate and iron nitrate solution UV-Visible absorbance spectrum is displayed in Figure 3. Cobalt nitrate peaks represent the visible region absorbance, and iron nitrate peaks are located in the UV region. The raw plant leaf extract absorbance presenting the bio-chemicals and their existence is expressed in the high absorbance peak in the UV region. The UV region peak denotes the high energy of electron molecules. Plant extract addition over the both nitrate solutions increased the electron mitigation, and the Co and Fe ions and their existing plant derivatives reduced the ions. Moreover, lattice oxygen with metal cations established the spinel metal ferrite structure.

The optical features and defects of the green synthesized CoFe_2_O_4_ nanoparticles were determined by UV-Visible spectroscopy. The absorbance peak of CoFe_2_O_4_ nanoparticles was located at 557 nm, which describes the UV region optical entity of CoFe_2_O_4_ nanoparticles, as shown in Figure 3. The UV region absorbance spectrum of CoFe_2_O_4_ nanoparticles denotes the enriched electron production over the surface [33]. The plant molecules’ interaction over the cobalt and iron source materials constructed the electron mitigation in the UV region. The bandgap of CoFe_2_O_4_ nanoparticles was calculated from the Kubelka–Munk relation [34,35], with the CoFe_2_O_4_ nanoparticles having a bandgap value of 2 eV (Figure 3b). This narrow bandgap value presents the highest e–h pair recombination activity and enhanced degradation behaviour [36]. The obtained bandgap of the CoFe_2_O_4_ nanoparticles demonstrated the narrow bandgap effect, which strongly affects organic pollutants.

### 3.4. VSM Analysis

**Figure 4:**
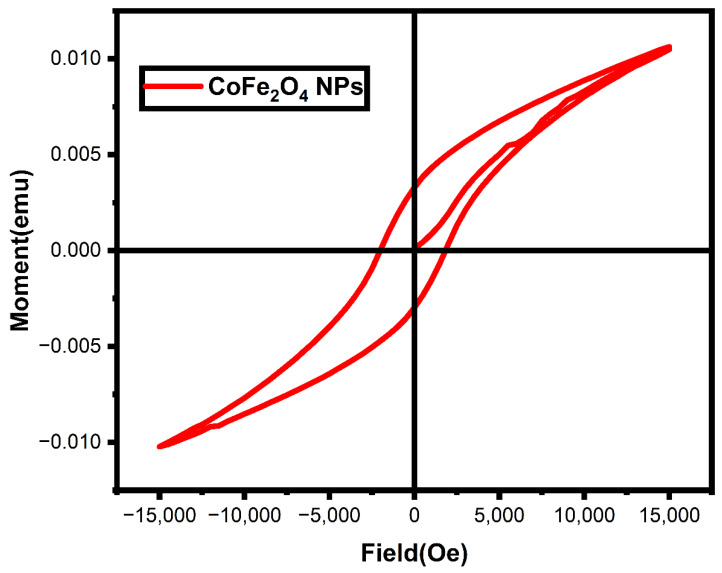
VSM spectrum of biosynthesized CoFe_2_O_4_ nanoparticles.

The magnetic behaviour of biosynthesized CoFe_2_O_4_ nanoparticles was characterized by VSM analysis, and the findings are presented in Figure 4. The M-H loop of biosynthesized CoFe_2_O_4_ nanoparticles displays the value of Saturation magnetization *M_S_* = 104.2 emu/g, coercive field *H_C_* = 1918.5 *Oe*, and retentivity *M_R_* = 3.1329 E^−3^ emu/g found in the sample. The obtained saturation magnetization is better than bulk cobalt ferrites (*M_S_* = 80 emu/g) [35]. The increased magnetization confirms the nanocrystalline nature and high magnetic field of the biosynthesized CoFe_2_O_4_ nanoparticles [36,37,38]. The small value of coercivity of the biosynthesized CoFe_2_O_4_ nanoparticles indicates that these nanoparticles are near the superparamagnetic limit. The M-H curve contains a linear part at higher fields, indicating a very significant paramagnetic contribution to this magnetization [39]. Based on the magnetic characteristics of the biosynthesized CoFe_2_O_4_ nanoparticles, they can be used for biomedical applications and should have increased catalytic efficiency against toxic organic effluents.

### 3.5. SEM with EDX

**Figure 5:**
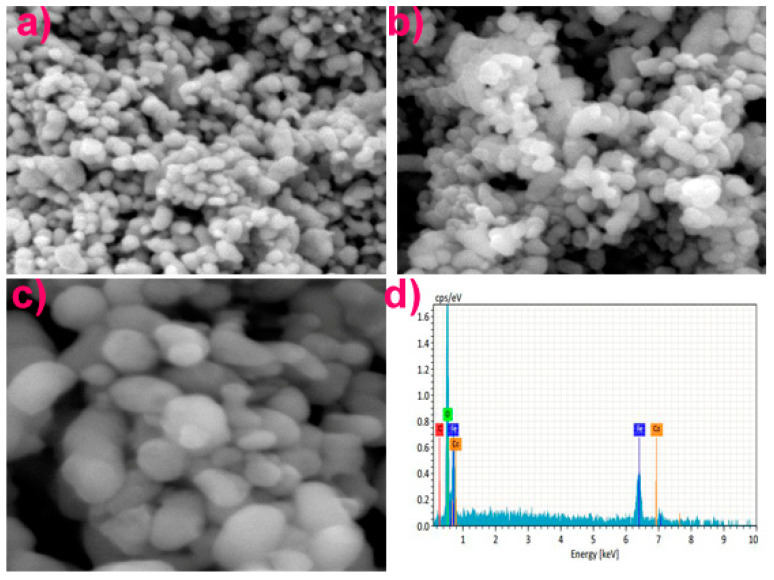
SEM images (**a**–**c**) and EDX (**d**) spectrum of biosynthesized CoFe_2_O_4_ nanoparticles.

The surface morphology and elemental presence of the biosynthesized CoFe_2_O_4_ nanoparticles were captured from SEM via EDX analysis. The different magnifications of the biosynthesized CoFe_2_O_4_ nanoparticles SEM images are presented in Figure 5a–c. The biosynthesized CoFe_2_O_4_ nanoparticles exhibited a poly-disperse spherical shape with even distribution over the surfaces. The spherically-shaped biosynthesized CoFe_2_O_4_ nanoparticles showed an enhanced surface area compared to other shapes [40,41,42,43]. The spherical shape explains the improved degradation of dye and organic pollutants. The biosynthesized CoFe_2_O_4_ nanoparticles’ existing materials are displayed in Figure 5d. The Co, Fe, and O elements confirm the synthesized title compounds. Based on SEM with EDX analysis, the biosynthesized CoFe_2_O_4_ nanoparticles evidence improved morphology.

### 3.6. TEM Analysis

**Figure 6:**
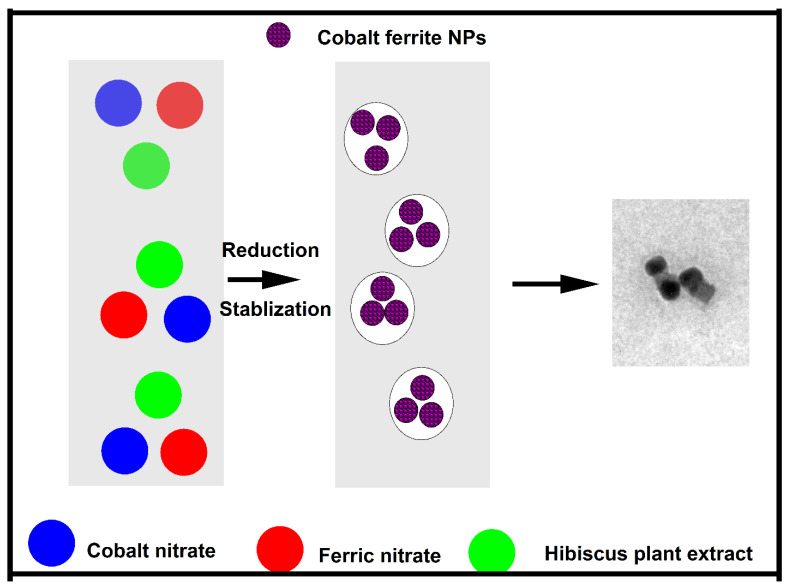
Nanostructure formation of biosynthesized CoFe_2_O_4_ nanoparticles.

**Figure 7:**
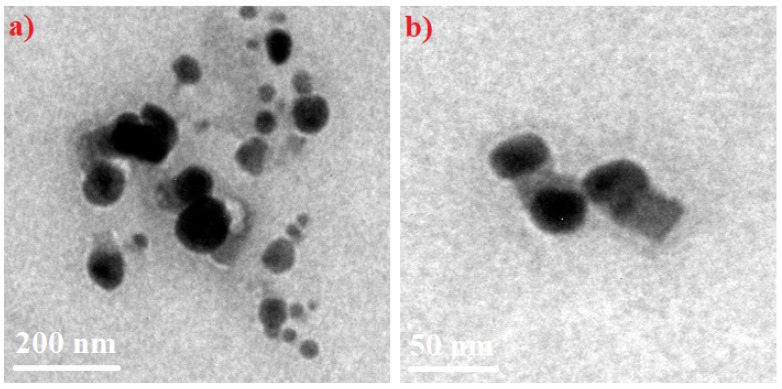
Different magnification ((**a**)-200 nm and (**b**)-50 nm) TEM images of biosynthesized CoFe_2_O_4_ nanoparticles.

The biosynthesized CoFe_2_O_4_ nanoparticles’ surface morphological characteristics were observed from TEM analysis. The biosynthesized CoFe_2_O_4_ nanostructure formation is shown in Figure 6 and their different magnified images are presented in Figure 7. The different magnifications of the biosynthesized CoFe_2_O_4_ nanoparticles exhibit their spherical shape as well as their distributions. The biomolecules adsorbed on the surface of the nanoparticles induce aggregation of nanoparticles, as displayed in Figure 7. The cobalt and iron elements are stabilized with lattice oxygen oriented from the bio-molecules. The spherical shape of the biosynthesized CoFe_2_O_4_ nanoparticles exhibits an improved surface area compared to other shapes of nanoparticles, and can provoke enhanced degradation activity [44,45,46,47]. The eventual distribution and sphericality of the biosynthesized CoFe_2_O_4_ nanoparticles increased the inactivation of both bacterial strains and organic compounds. The size of the biosynthesized CoFe_2_O_4_ nanoparticles is 25 nm, which is very close to crystallite size, and their electron accumulation was ensured by UV-Vis analysis.

### 3.7. Photocatalytic Activity

**Figure 8:**
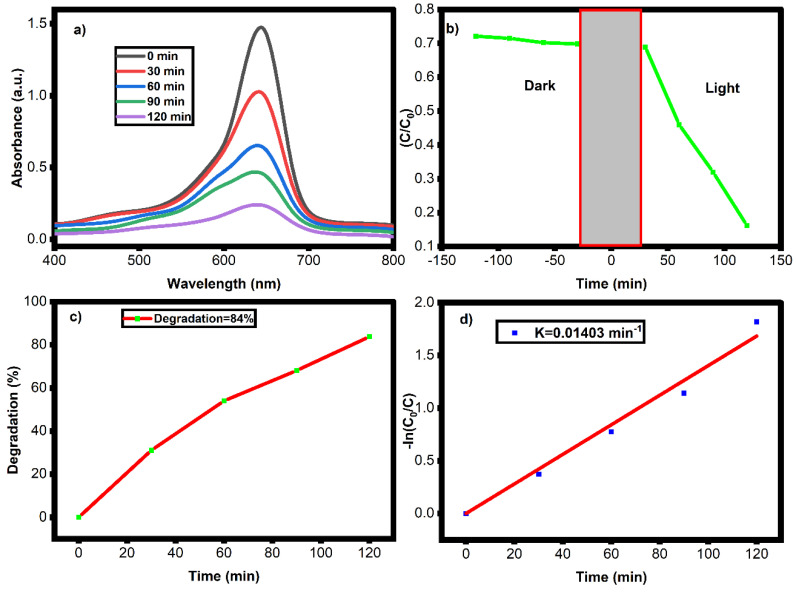
(**a**) Photocatalytic degradation, (**b**) light kinetics, (**c**) degradation percentage, and (**d**) pseudo-order kinetic spectrum of biosynthesized CoFe_2_O_4_ nanoparticles.

**Figure 9:**
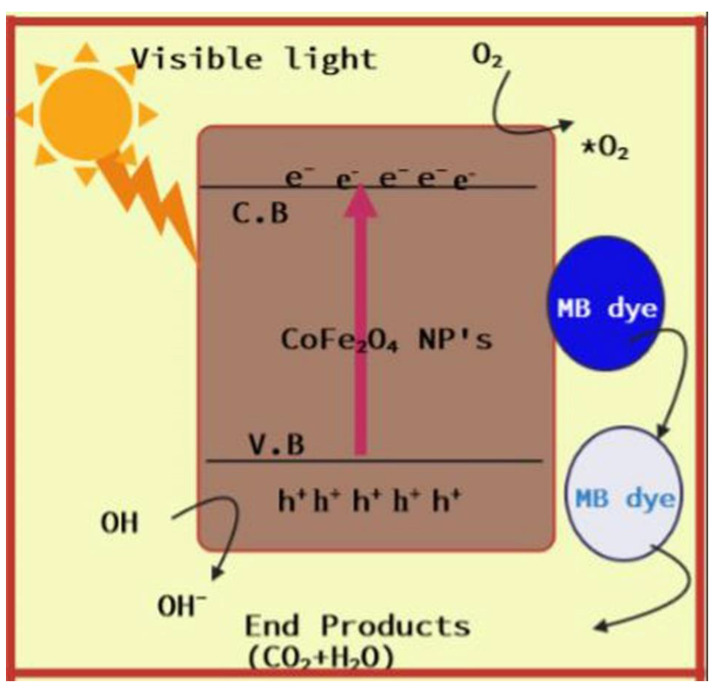
Photocatalytic dye degradation mechanism of biosynthesized CoFe_2_O_4_ nanoparticles.

The organic dye pollutant MB was used to evaluate the catalytic efficiency of the green synthesized CoFe_2_O_4_ nanoparticles under visible light irradiation. The absence of light MB dye absorbance was high, and it decreased periodically for both light and time. The decreased absorbance determined the n–π* transition of the organic MB dye (Figure 8). The degradation of MB dye is evident from the dislocation of peaks and decreased absorbance. The decreased absorbance intensity of the MB dye displays its disintegration. The absorbance of the dye solution was measured every 30 min; 120 min of light irradiation over the catalyst surface achieved 84% degradation, and in the absence of light their degradation was much less compared to with light (Figure 9). Magnetic nanoparticles and magnetic ferrites showed enhanced catalytic degradation and microbial inactivation due to their suppressed electron-hole pair, high biocompatibility, generated reactive oxygen species, and bio-degradability. The light irradiation on the catalyst solution excited the electrons from the valence band to the conduction band. The holes in the excited electrons create e–h pair recombination activity [48,49]. The generated holes produce the oxidation property, and the electrons produce the reduction property, which forms super-oxides and hydroxyl radicals. These radical and super-oxides dissociate the organic dye compounds and convert it to non-toxic small molecules such as CO_2_ and H_2_O.

CoFe_2_O_4_ + light → holes (valence band) + electrons (conduction band)

e^−^ + O_2_ → *O_2_^−^

h^+^ + H_2_O → OH

*O ^⋅^^−2^ + MB → Dissociated dye molecules

·OH + MB → Dissociated dye molecules

h^+^ + MB → Dissociated dye molecules

The photocatalytic degradation is influenced by various factors, such as (i) light source, (ii) dye solution, (iii) pH, and (iv) catalyst concentration. These factors are defined as the catalytic degradation of the samples and their tabulation is listed in Table 1. The cobalt ferrite-associated nanoparticles revealed better catalytic activity in UV light irradiation due to OH radical formation [50,51,52,53]. The pure cobalt ferrite nanoparticles under sunlight illumination exhibited superior degradation performance against various dyes [51,52,53,54,55]. The different dopants against the cobalt ferrites induced higher surface area and extended e–h pair recombination, increasing the degradation potential against toxic dye and organic pollutants [53,54,55,56,57]. The present work was carried out by visible light irradiation, narrow bandgap, lowset crystallite sizes, and spherically-shaped nanostructure conditions. With these conditions, CoFe_2_O_4_ nanoparticles revealed better degradation potency than the above-mentioned work. The present work achieved enhanced dye degradation efficiency without using any dopant, and the nanostructure was achieved via green manufacturing.

#### Comparison to P25 Catalyst

**Figure 10:**
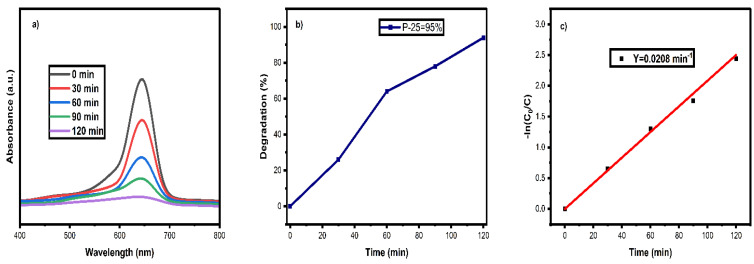
(**a**) P25 Photocatalyst degradation spectrum, (**b**) degradation efficiency graph, and (**c**) kinetic spectrum.

The biosynthesized CoFe_2_O_4_ nanoparticles were compared with a commercial photocatalyst, namely, P25 (TiO_2_) particles. The P25 photocatalyst exhibited 95% degradation over 120 min of visible light irradiation (Figure 10). The commercial P25 photocatalyst was able to highly decompose the dye solution and break its molecular bonding thanks to high photo-response, adsorption, and charge separation activities [58,59,60,61]. The biosynthesized CoFe_2_O_4_ nanoparticles achieved nearly equal results to the degradation ability of the commercial P25 photocatalysts.

### 3.8. Antibacterial Activity

**Figure 11:**
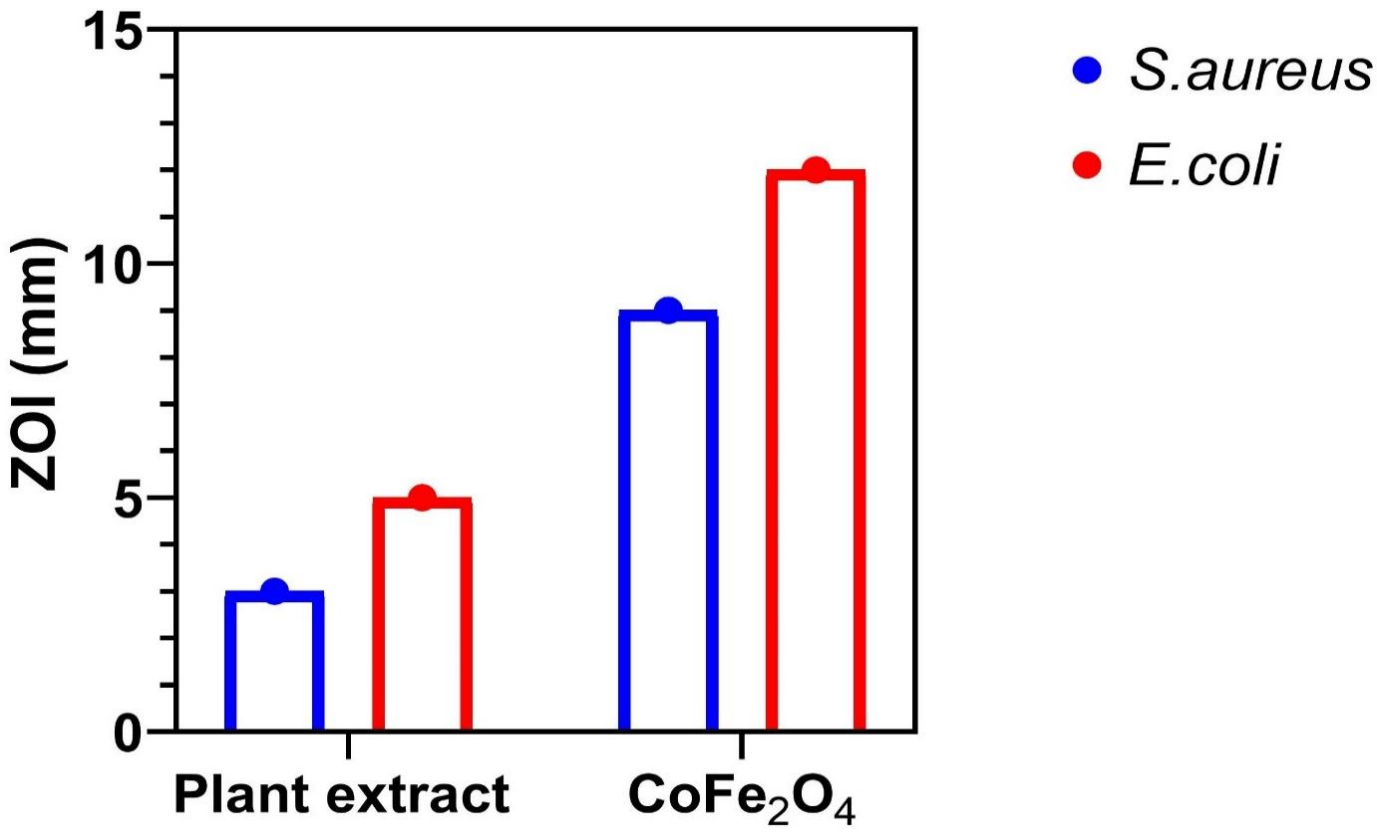
Antibacterial activity of biosynthesized CoFe_2_O_4_ nanoparticles.

The antibacterial activity of plant extract and biosynthesized CoFe_2_O_4_ nanoparticles was monitored against *S. aureus* and *E. coli* bacterial strains, with their zone of inhibitions (ZOI) displayed in Figure 11. Gram-negative bacteria showed more inactivation than gram-positive bacteria. Plant biomolecules restricted cell viability and reduced the security of the cell system. The incorporation of plant extract in the biosynthesized CoFe_2_O_4_ nanoparticles increased the bioactivity. The metal ions contact the bacterial cells, which disrupts cell protection and produces reactive oxygen species and oxidative stress on the bacterial surfaces. The spinel Co and Fe cause increased DNA fragmentation, and their diffused ions interact with the cell membrane. The cell membrane’s interaction with the biosynthesized CoFe_2_O_4_ nanoparticles allows the cobalt and iron ions into the cell system. The ferrite ions enter into the cell and disrupt the its regular activities, leading to the inactivation state [62,63,64,65,66,67]. The plant extract and spinel ferrite biosynthesized CoFe_2_O_4_ nanoparticles revised the cell membrane and DNA and protein actions within the cell system. These modifications to the cell system can provoke cell inactivation and boost cell death.

## 4. Conclusions

The current work investigated the bio-production of CoFe_2_O_4_ nanoparticles from *Hibiscus rosa sinensis* plant leaf. The leaf extract provokes the reduction and stabilization of biogenic CoFe_2_O_4_ nanoparticles, enhancing the structural, optical, and magnetic properties of the spinel ferrite CoFe_2_O_4_ nanoparticles. The improved crystallite size, bandgap, and super-paramagnetization increase e–h pair recombination and radical generation, and the spinel ferrite structure of the CoFe_2_O_4_ nanoparticles obtains high energy separation activity. Moreover, biogenic nanoparticles produce chemical-free output, and their derivatives are not implicated in contamination activities. The obtained values demonstrate that CoFe_2_O_4_ nanoparticles are enhanced in terms of their degradation power towards toxic compounds, and their biogenic nano-production promotes chemical-free nanoparticle production. Based on the attained values, CoFe_2_O_4_ nanoparticles can be used in water remediation and the instrument development process.

## Figures and Tables

**Table 1:** Photocatalytic comparison table of biosynthesized CoFe_2_O_4_ nanoparticles.

S.No.	Nanomaterials	Dye	Time	Light	Degradation	Reference
1.	MxCo1-xFe_2_O_4_ NPs	Methylene Blue	100 min	UV-light irradiation	96.0%	[50]
2.	TiO_2_/CoFe_2_O_4_	Procion Red MX-5B	300 min	UV illumination	95%	[51]
3.	CoFe_2_O_4_	RhB	150 min	sunlight irradiation	68%	[52]
4.	CoFe_2_O_4_	CR	150 min	sunlight irradiation	96%	[52]
5.	CoFe_2_O_4_	MO	150 min	sunlight irradiation	87%	[52]
6.	CoFe_2_O_4_	MB	150 min	sunlight irradiation	81%	[52]
7.	CoFe_2_O_4_	4-N	150 min	sunlight irradiation	63%	[52]
8.	CoFe_2_O_4_	4-C	150 min	sunlight irradiation	55%	[52]
9.	CoFe_2_O_4_	Carbamazepine	100 min	Hg lamp	80%	[53]
10.	Co1-xZnxFe_2_O_4_ a	Rhodamine B	210 min	Halide lamp	99.9%	[54]
11.	CoFe_2_O_4_/ZnOa	Phenolphthalein	45 min	UV-lamp	89%	[55]
12.	CoFe_2_O_4_/AgBr	Methyl orange	60 min	LED lamp	89%	[56]
13.	CoFe_2_O_4_@methycellulose	Metronidazole	120 min	UV lamp	85.3%	[57]

## Data Availability

All research data are provided in the manuscript.
